# Nopal Cactus (*Opuntia ficus-indica*) as a Source of Bioactive Compounds for Nutrition, Health and Disease

**DOI:** 10.3390/molecules190914879

**Published:** 2014-09-17

**Authors:** Karym El-Mostafa, Youssef El Kharrassi, Asmaa Badreddine, Pierre Andreoletti, Joseph Vamecq, M’Hammed Saïd El Kebbaj, Norbert Latruffe, Gérard Lizard, Boubker Nasser, Mustapha Cherkaoui-Malki

**Affiliations:** 1 Laboratoire Bio-PeroxIL, EA7270 University of Bourgogne, 6 Bd Gabriel, Dijon F-21000, France; 2 Laboratoire de Biochimie et Neurosciences, Faculté des Sciences et Techniques, Université Hassan I, BP 577, Settat 26 000, Morocco; 3 Inserm and HMNO, CBP, CHRU Lille, Lille 59037, France; 4 Laboratoire de recherche sur les Lipoprotéines et l’Athérosclérose, Faculté des Sciences Ben M’sik, Avenue Cdt Driss El Harti BP. 7955, Université Hassan II-Mohammedia-Casablanca, Casablanca 20 000, Morocco; 5 Inserm and Laboratoire Bio-PeroxIL, EA7270 University of Bourgogne, 6 Bd Gabriel, Dijon F-21000, France

**Keywords:** anti-inflammatory, antioxidants, cell signaling, *Opuntia ficus-indica*, polyphenols

## Abstract

*Opuntia ficus-indica*, commonly referred to as prickly pear or nopal cactus, is a dicotyledonous angiosperm plant. It belongs to the *Cactaceae* family and is characterized by its remarkable adaptation to arid and semi-arid climates in tropical and subtropical regions of the globe. In the last decade, compelling evidence for the nutritional and health benefit potential of this cactus has been provided by academic scientists and private companies. Notably, its rich composition in polyphenols, vitamins, polyunsaturated fatty acids and amino acids has been highlighted through the use of a large panel of extraction methods. The identified natural cactus compounds and derivatives were shown to be endowed with biologically relevant activities including anti-inflammatory, antioxidant, hypoglycemic, antimicrobial and neuroprotective properties. The present review is aimed at stressing the major classes of cactus components and their medical interest through emphasis on some of their biological effects, particularly those having the most promising expected health benefit and therapeutic impacts.

## 1. Introduction

*Opuntia ficus-indica* *(L.) Mill.*, commonly called prickly pear or nopal cactus, belongs to the dicotyledonous angiosperm *Cactaceae* family, a family that includes about 1500 species of cactus. *O. ficus indica* is a tropical and subtropical plant. It can grow in arid and semi-arid climates with a geographical distribution encompassing Mexico, Latin America, South Africa and Mediterranean countries [1]. Because the oldest Arabic medicine treatises do not mention cactus, it is generally accepted that Spain might have introduced the nopal fig tree in the 15th century from Central America after the conquest of the northwest of Africa. 

Nowadays, local populations in Morocco distinguish three varieties of *O. ficus indica*. The first, with prickly cladodes, is called “Christians’ nopal” and is commonly used as a field fence. The second, with inermis cladodes, is “Muslims’ nopal” and serves as a green fodder for cattle. The last variety, with large inermis cladodes, is referred to as “Moses’ nopal”, grows essentially in the south of Morocco (Ifni region) and produces a big pear. 

Nopal cactus is employed in health, nutrition and cosmetics in the forms of tea, jam, juice and oil extracted from prickly pear seeds. It is used as a herbal remedy for diverse health problems in different countries. For instance, in the sub-Saharan traditional medicine pharmacopeia, cactus flowers and fruits are given as anti-ulcerogenic or antidiarrheal agents; flowers being also administered as an oral anti-hemorrhoid medication and cladode sap as a treatment for whooping cough. On the other hand, indigenous populations consume substantial amounts of either fresh or dry fruits as food. In these populations, cactus cladodes, fruits and flowers are featured for their interesting contents of antioxidants, pectin polysaccharides and fibers. 

Recent scientific reports have highlighted the presence of natural cactus molecules, which may have high potential interest in human health and medicine [2,3,4]. As a general rule in herbal medicine, the extraction of bioactive compounds from permeable solid plant materials using solvents constitutes a key step in the manufacture of phytochemical-rich products. *Opuntia ficus indica* is known for its high content in polyphenols exhibiting antioxidant and anti-inflammatory properties [1,5]. Interestingly, alkaloids, indicaxanthin, neobetanin, and various flavonoids have been isolated from the cactus [6], along with polysaccharides which are abundant in cladode extracts and endowed with antidiabetic and antiglycation effects [7].

This review is dedicated to recent developments in the area of medically relevant compounds isolated from each of the different aerial parts (cladodes, flowers and fruits) of *Opuntia ficus-indica,* and to the different usages of the cactus in human foods, health promotion, disease prevention and therapy.

## 2. General Compound Content of Cactus

Cactus fruit contains substantial amounts of ascorbic acid, vitamin E, carotenoids, fibers, amino acids and antioxidant compounds (phenols, flavonoids, betaxanthin and betacyanin) which have been put forward to account for its health benefits such as hypoglycemic and hypolipidemic action, and antioxidant properties [8,9,10]. Several reports have documented the abundance of vitamins and minerals in cactus [11]. In this respect, the fruit of *O. ficus indica* is a valuable source of nutrients [12] as well as antiulcerogenic [13,14], antioxidant [5,13,14,15,16], anticancer [16], neuroprotective [17], hepatoprotective [18], and antiproliferative [19] compounds. *Opuntia ficus indica* flowers contains different flavonoids notably kaempferol and quercetin [20]. Cactus peel and seeds can be used to prepare cactus oil, peel lipids being enriched in essential fatty acids and liposoluble antioxidants [21]. The cactus cladodes contain vitamins, antioxidants and various flavonoids, particularly quercetin 3-methyl ether, a highly efficient radical scavenger [22,23]. Cladodes of *O. ficus-indica* extracts may lower cholesterol level and convey antiulcer and anti-inflammatory mechanisms, and the water extract remarkably improves wound healing [14,24]. 

## 3. Individual Classes of Cactus Compounds and Related Biological Activities

Regarding its composition in polyphenols, vitamins and other specific compounds, the cactus pear appears to be an excellent candidate for nutritional diet recommendations and therapeutic indications. The spectrum of biological and medical effects reported for each class of cactus compounds is presented thereafter.

### 3.1. Phenolic Compounds

Polyphenols represent a family of organic molecules widely distributed in the plant kingdom. As suggested by their name, their chemical structures are characterized by the presence of several phenolic groups, which may be associated with more or less complex groups of chemicals, generally of high molecular weight. These compounds are usually byproducts of plant metabolism. The growing interest in polyphenols results from their antioxidant potential, which is involved in health benefits such as the prevention of inflammation [24], cardiovascular dysregulation and neurodegenerative diseases. Polyphenols have also proven anticancer activity. 

All parts of the cactus plant are rich in members of the polyphenol family such as various flavonoids and phenolic acids (Table 1). In the flower, gallic acid and 6-isorhamnetin 3-*O*-robinobioside are the major compounds, amounting to 4900 and 4269 mg/100 g of dry matter, respectively [20,25,26,27]. Other phenolic molecules are present in small quantities not exceeding 10 mg/g (Table 2). In the fruit pulp, total phenol content is 218.8 mg/100 g [28], along with a high content of isorhamnetin glycosides (50.6 mg/100 g) compared to other flavonoids [14,29,30,31,32]. Fruit seeds contain high amounts of phenolic compounds ranging from 48 to 89 mg/100 g and including feruloyl derivatives, tannins and sinapoyl diglucoside [33] (Table 1). Interestingly, fruit peel has a very high phenol content of 45.7 g/100 g. Several of these phenols are bioactive molecules, notably flavonoid derivatives such as kaempherol and quercetin, the contents of which are 0.22 and 4.32 mg/100 g, respectively [5,30,34]. Cactus flowers appear to be the most important source of polyphenols and flavonoids.

Interestingly, some polyphenols are produced only by cladodes of some varieties of cactus such as the snowshoeing cactus. This plant presents high amounts of unusual flavonoid-like compounds such as nicotiflorin (146.5 mg/100 g) and narcissin (137.1 mg/100 g) (Table 1) along with high content values found for isoquercetin and ferulic acid: 39.67 and 34.77 mg/100 g, respectively [4,29,35,36,37]. Cladode age, environment, soil type and climate could explain these variations in cactus polyphenol contents. 

**Table 1:** Distribution and contents of phenols and flavonoids in the various parts of *O. ficus-indica*.

Plant tissue	Main Component Identified	Content in mg/100 g	References
**Flower**	Gallic acid	1630–4900	[20,25,26,27]
	Quercetin 3-*O*-Rutinoside	709	
	4 Kaempferol 3-*O*-Rutinoside	400	
	5 Quercetin 3-*O*-Glucoside	447	
	6 Isorhamnetin 3-*O*-Robinobioside	4269	
	7 Isorhamnetin 3-*O*-Galactoside	979	
	8 Isorhamnetin 3-*O*-Glucoside	724	
	9 Kaempferol 3-*O*-Arabinoside	324	
**Pulp**	Total phenolic acid	218.8	[13,28,29,31,32,38]
	Quercetin	9	
	Isorhamnetin	4.94	
	Kaempferol	0.78	
	Luteolin	0.84	
	isorhamnetin glycosides	50.6	
	Kaempferol	2.7	
**Seed**	Total phenolic acid	48–89	[33]
	Feruloyl-sucrose isomer 1	7.36–17.62	
	Feruloyl-sucrose isomer 2	2.9–17.1	
	Sinapoyl-diglucoside	12.6–23.4	
	Total Flavonoids	1.5–2.6	
	Total Tannins	4.1–6.6	
**Skin fruits**	Total phenolic acid	45,700	[5,30,34]
	Total Flavonoid	6.95	
	Kaempferol	0.22	
	Quercetin	4.32	
	Isorhamnetin	2.41–91	
**Cladode**	Gallic acid	0.64–2.37	[4,29,35,36,37]
	Coumaric	14.08–16.18	
	3,4-dihydroxybenzoic	0.06–5.02	
	4-hydroxybenzoic	0.5–4.72	
	Ferulicacid	0.56–34.77	
	Salicylicacid	0.58–3.54	
	Isoquercetin	2.29–39.67	
	Isorhamnetin-3-*O*-glucoside	4.59–32.21	
	Nicotiflorin	2.89–146.5	
	Rutin	2.36–26.17	
	Narcissin	14.69–137.1	

Health beneficial effects of cactus polyphenols might be conditioned by their antioxidant and radical scavenging activities. For instance, gallic acid, largely found in cactus flowers, exhibits high antioxidant activity responsible for its ability to reduce DNA damage [39] and to buffer free radicals [40]. At a concentration of 4.17 mM, it may neutralize 44% of 2,2-diphenyl-1-picrylhydrazyl radical and 60% of hydrogen peroxide in given experimental conditions. Gallic acid also exerts a cytotoxic activity against tumoral cells from leukemia, lung and prostate cancer origins [41].

*Opuntia ficus-indica* cladodes are rich in nicotiflorin which, through antiinflammatory and neuroprotective mechanims, was shown to reduce brain infarct size, to attenuate neurological deficits induced by ischemia, and to up-regulate endothelial nitric oxide synthase in cultured rat brain vascular endothelial cells [42]. Nicotiflorin is neuroprotective against hypoxia-, glutamate- or oxidative stress-induced retinal ganglion cell death at nanomolar concentrations [43]. In a murine multi-infarct dementia model, nicotiflorin preserved spatial memory performances measured in Morris water maze tests. Besides this protective effect on memory dysfunction, nicotiflorin also protects against energy metabolism failure and oxidative stress. In ischemic brains, these beneficial effects were associated with attenuation of rises in lactic acid and malondialdehyde (MDA) and with prevention of drops in lactate dehydrogenase (LDH), Na^+^K^+^ATPase, Ca^2+^Mg^2+^ATPase and superoxide dismutase (SOD) activities [44].

As mentioned above, the fruit peel contains large amounts of isorhamnetin. Isorhamnetin (3'-methoxy-3,4',5,7-tetrahydroxyflavone) exerts anticancer action by inhibition of epidermal growth factor (EGF)-induced neoplastic cell transformation through a direct lowering of MAP (mitogen-activated protein)/ERK (extracellular signal regulated kinase) kinase 1 and phosphoinositol 3-kinase signaling pathways [45]. Isorhamnetin exhibits cardioprotective effects by improving viability of neonatal rat ventricular myocytes under *in vitro* ischemia/reperfusion (I/R) *via* inhibition of lactate dehydrogenase (LDH) activity and prevention of apoptosis [46]. Isorhamnetin improves skin barrier function through activation of Peroxisome Proliferator-Activated Receptor (PPAR)-α and suppression of inflammatory cytokines production [47]. It also inhibits adipocyte differentiation of murine 3T3 fibroblasts *via* a decrease of adiponectine expression and secretion, and downregulation of mRNAs of PPAR-γ and C/EBP-α, the major adipogenic nuclear receptors [48]. In contrast, isorhamnetin significantly increases the expression of PPAR-γ in tumor tissues obtained from xenograft model of gastric cancer cells and, in combination with chemotherapeutic drugs, causes strong antiproliferative effects and cytotoxicity [49]. These different effects of a same PPAR ligand are explained by the fact that a ligand-activated nuclear receptor can exert different biological activities through the recruitment of their coregulator partners in a way specific to the cell type context.

### 3.2. Fatty Acids

Chromatographic analyses of total lipids extracted from cactus cladodes (Table 2) [50] show that palmitic acid (C16:0), oleic acid (C18:1), linoleic acid (C18:2), linolenic acid (C18:3) contribute 13.87, 11.16, 34.87 and 32.83% of the total fatty acid content, respectively (Table 1). These four fatty acids thus represent over 90% of total fatty acids with linoleic and linolenic acids, the major polyunsaturated fatty acids, amounting to 67.7%. The linoleic acid content in cactus cladode (34.87%) is thus close to the percentage (29% to 40.41%) found in argan oil [51]. It is however lower than in extracts from barely (51.26%) and soybean (53.0%), respectively (Table 2) [50].

**Table 2:** Comparison of the fatty acid composition of *O. ficus-indica* and other edible oils Compositions are expressed in g/100 g fatty acids.

Fatty Acid	C12:0	C14:0	C16:0	C16:1	C18:0	C18:1	C18:2	C18:3	C20:0	C22:0	C22:1	C24:0	Reference
Cladode	1.33	1.96	13.87	0.24	3.33	11.16	34.87	33.23	-	-	-	-	[50]
Cactus seed oil	-	-	20.1	1.80	2.72	18.3	53.5	2.58	-	-	-	-	[52]
Cactus seed oil	-	-	9.32	1.42	3.11	16.77	70.29	nd	-	-	-	-	[53]
Fruits pulp oil	-	1.13	34.4	1.62	2.37	10.8	37	12.68	-	-	-	-	[52]
Prickly pear peel	0.71	1.95	23.1	2.48	2.67	24.1	32.3	9.27	nd	0.5	-	0.41	[21]
Argan oil	-	0.10	11.7	0.14	4.9	36.6	31.3	0.09	0.33	0.12	-	0.06	[54]
Olive oil	-	11.5	0.9	1.4	61.9	3.8	1.1	0.23	-	-	-	-	[55]
Grape seed oil	-	0.06	8.3	0.1	3	12	67.6	0.3	0.2	0.1	0.02	0.01	[56]
Soybean oil	-	-	6	0.4	2.2	26.1	50.1	14.5	-	-	-	-	[57]
Corn oil	-	-	13.4	Traces	1.5	27.4	56	0.9	0.2	-	-	-	[58]
Sunflower oil	-	0.08	7.4	0.09	4.56	25.17	60.15	0.3	-	-	-	0.34	[59]

Several studies have indicated that cactus particularly; fruits, pulp, seed and pickely pear peel were rich in linolenic, oleic and palmitic acids [21,52,53,60]. High level of omega-6 linoleic acid was reported in cactus seed oil (53.5% to 70.29%) (Table 1), and this level is higher than in sunflower oil [59], grape seed oil or sesame oil. As a precursor of arachidonic acid, linoleic acid has long been accepted as having a hypocholesterolemic effect and inhibitory properties against colon cancer metastatic cells [61]. Omega-3 linolenic acid is known to be beneficial for health, cardiovascular diseases, inflammatory conditions, autoimmune disorder and diabetes.

### 3.3. Vitamins

The *Opuntia ficus indica* develops a fruit known as cactus pear, a fleshy bay (pulp) containing seeds and enveloped by a prickly peel (skin). The fruit, particularly its skin, is enriched in vitamin E at amounts up to 17.6 g/kg of α-tocopherol (Table 3). In contrast, the oil extracted from the fruit’s seeds has a low content in vitamin E: 0.403 g/kg, mostly γ-tocopherol (0.330 g/kg) [21,52,60]. Such an amount is very low compared to argan oil content (7.6 to 8.6 g/kg) [62,63]. The essential oil extracted from the fruit’s pulp is rich in σ-tocopherol with 4.220 g/kg (Table 3). Cactus pear contains 180 to 300 mg/kg of vitamin C. This content is higher than that found in other common fruits like apple, banana, or grape [64]. Vitamin K1 is present in all parts of the fruit, ranging from 0.5 to 1 g/kg [52,60]. Vitamin B is present only in the cladodem in which it is found in trace amounts [65]. To our knowledge, the precise vitamin contents in flowers of *Opuntia ficus indica* still remains to be elucidated. 

**Table 3:** Distribution and contents of vitamins in the different parts of fruit and cladode from prickly pear of *O. ficus-indica.* Vitamin contents are expressed as mg/100 g tissue.

	Pulp	Seeds	Skin	Cladode	Source
Vitamin K1	53.2	52.5	109	----	[5,21,28,31,32,60,66]
Vitamin C,	34–40	----	----	7–22	
Vitamin B1	----	----	----	0.14	
Vitamin B2	----	----	----	0.60	
Vitamin B3	----	----	----	0.46	
α-Tocopherol,	84.9	56	1760	----	
β-Tocopherol,	12.6	12	222	----	
γ-Tocopherol,	7.9	33	174	----	
σ-Tocopherol	422	5	26	----	
Total vitamin E	527.4	106	2182	----	

### 3.4. Sterols

Ramadan and Morsel [52,60], have documented β-sitosterol as the major sterol extracted from different parts of the fruit oils: pulp, skin and seeds, with a content ranging from 6.75 to 21.1 g/kg [52,60]. Campesterol is present in the pulp, seed and skin, in an amount of 1.66 to 8.76 g/kg (Table 4). Similar contents of campesterol are found in some other food oils such as argan oil (4 g/kg) [67], whereas higher contents have been measured in soybean oil (between 19 and 23 g/kg) [67]. Other sterols are found in small quantities notably stigmasterol, lanosterol, avenasterol Δ^5^,Δ^7^-avenasterol, Δ^7^-avenasterol and ergosterol, So far, sterol composition of the essential oil of cladode and flowers remains to be determined. In comparison with cactus, in argan oil, for instance, sterols such as spinasterol and schottenol have been identified [67].

**Table 4:** Distribution and contents of sterols in the various parts of the *O. ficus-indica* fruit including pulp, seeds and skin. Sterol contents are expressed in g/kg.

Main Component Identified	Pulp	Seed	Skin	References
Campesterol	8.74	1.66	8.76	[21,60]
Stigmasterol	0.73	0.30	2.12	
Lanosterol	0.76	0.28	1.66	
β-Sitosterol	11.2	6.75	21.1	
Δ^5^-Avenasterol, Δ^7^-Avenasterol	1.43	0.29	2.71	
Δ^7^-Avenasterol	----	0.05	----	
Ergosterol	----	----	0.68	

### 3.5. Mineral Compounds

Cactus fruit’s seeds are rich in minerals, with a predominance of potassium and phosphorus at 163 and 152 mg/100 g (Table 5), respectively. Remarkable also is the presence of large quantities (given in mg/100 g) of magnesium (74.8), sodium (67.6) and calcium (16.2) (Table 5) [68,69,70]. In cladode, the major minerals are potassium and calcium, with amounts ranging from 235 to 5520 mg/100 g (Table 5) [65,71,72]. In pulp, potassium is present at 161 mg/100 g, exceeding the concentration of other minerals like calcium and magnesium (Table 5) [70,73].

**Table 5:** Distribution and contents of minerals in the various parts of *O. ficus-indica*. Mineral contents are expressed as mg/100 g.

Main component identified	Pulp	Seed	Cladode	References
Calcium	27.6	16.2	5.64–17.95	[65,66,70,71,72,74]
Calcium oxalate	----	----	11.5 to 4.3	
Magnesium	27.7	74.8	8.80	
Sodium	0.8	67.6	0.3–0.4	
Potassium	161	163	2.35–55.20	
Iron	1.5	9.45	0.09	
Phosphorus	----	152	0.15–2.59	
Zinc	----	1.45	0.08	
Copper	----	0.32	----	
Manganese	----	Trace	0.19–0.29	

### 3.6. Amino Acids

In cactus cladodes, the major amino acid detected is glutamine, followed by leucine, lysine, valine, arginine, phenylalanine and isoleucine. By contrast, in cactus seed the major amino acid is glutamic acid at a percentage varying from 15.73% to 20.27%, followed by arginine, (4.81% to 14.62%) (Table 6) [75,76]. Interestingly, in the cactus fruit, the two predominant amino acids are proline and taurine, which represent 46% and 15.78% of the total amino acid content, respectively. Total proteins in fruit seeds (13.62%) are higher than in cladodes (4%–10%) (Table 6) [75,76]. Thus, fruit seeds and pulp can be considered as very good sources of amino acids and proteins [75,76,77].

**Table 6:** Distribution and contents of amino acids content in seeds, cladode and fruit juice from *O. ficus-indica*. Amino acid contents are expressed as g/100 g.

Amino Acid	Cladode	Fruit	Seeds	References
Alanine	1.25	3.17	4.75	[65,73]
Arginine	5.01	1.11	6.63	
Asparagine	3.13	1.51	Trace	
Asparaginic acid	4.38	Trace	10.42	
Glutamic acid	5.43	2.40	21.68	
Glutamine	36.12	12.59	Trace	
Cystine	1.04	0.41	0.37	
Histidine	4.18	1.64	3.11	
Isoleucine	3.97	1.13	6.20	
Leucine	2.71	0.75	9.94	
Lysine	5.22	0.63	6.79	
Methionine	2.92	2.01	0.70	
Phenylalanine	3.55	0.85	5.25	
Serine	6.68	6.34	8.46	
Threonine	4.18	0.48	1.53	
Tyrosine	1.46	0.45	3.09	
Tryptophane	1.04	0.46	Trace	
Valine	7.72	1.43	6.02	
α-Aminobutyric acid	Trace	0.04	Trace	
Carnosine	Trace	0.21	Trace	
Citrulline	Trace	0.59	Trace	
Ornithine	Trace	Trace	Trace	
Proline	Trace	46.00	Trace	
Taurine	Trace	15.79	Trace	
Glycine	Trace	Trace	5.06	

## 4. Cactus and Compounds in Nutritional and Medical Practice

### 4.1. Cactus in Nutrition and Prevention of Disease

**Figure 1:**
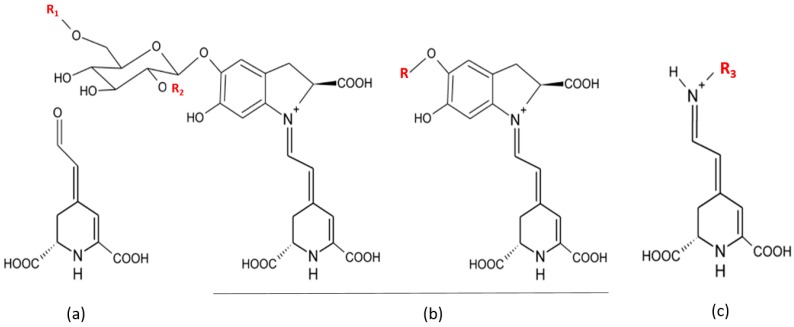
General structure of betalamic acid (**a**), betacyanins (**b**) and betaxanthins (**c**) [81].

The nutritional value of cactus pear fruit mainly rests on its content in ascorbic acid, vitamin E, carotenoids, fibers, amino acids, and on large amounts of glucose and fructose. Prickly pears are also rich in phenols, flavonoids, betaxanthins and betacyanins (Figure 1 and Table 7), which favor a healthy status through hypoglycaemic and hypolipidemic actions, and antioxidant properties [4,8,9,10]. Remarkably, among existing natural pigments betalains are present at high amount in cactus. Regarding consumers’ increasing aversion for synthetic colorants, natural colorants, such as the red betacynins and the yellow betaxanthins, represent a good natural alternative to meet the growing demand of the food industry. The antioxidant properties of these betalain pigments represent an additional argument in favor of the development of their use in nutrition and health [78,79,80].

**Table 7:** Names of betaxanthins and betacyanins present in *Opuntia spp*.

Compounds	Name	Radical	*Opuntia* Specie	References
Betaxanthins	Portulacaxanthin I	R3 = hydroxyproline	*O. ficus-indica*	[78]
	Portulacaxanthin III	R3 = glycine	*O. ficus-indica*	[78,81]
	Muscaaurin	R3 = histidine	*O. robusta*, *O. ficus-indica*, *O. megacantha*	[78,82]
	Indicaxanthin	R3 = proline	*O. robusta Wendl, O. robusta, O. streptacantha Lemaire, O. ficus-indica, O. megacantha, O. albi-carpa*	[12,78]
	(*S*)-serine-betaxanthin	R3 = serine	*O. ficus-indica*	[78,81]
	(*S*)-valine-betaxanthin	R3 = valine	*O. ficus-indica*	[81]
	(*S*)-isoleucine-betaxanthin	R3 = isoleucine	*O. ficus-indica*	[81]
	γ-Aminobutyric acid-Bx	R = butyric acid	*O. spp*	[82]
	Methionine-betaxanthin	R3 = methionine	*O. spp*	[82]
	(S)- Phenylalaine-betaxanthin	R3 = phenylalaine	*O. ficus-indica*	[81]
	Vulgaxanthin I	R3 = glutamine	*O. robusta Wendl, O. ficus-indica*	[81]
	Vulgaxantin Ii	R = glutamic acid	*O. streptacantha; O. beta vulgaris L.spp. V. Pablo; O. bergeriana; O. ficus indica; O. alba-carba; O. robusta and O. Spp*	[82]
	Vulgaxantin III	R = asparagine	*O. streptacantha; O. beta vulgaris L.spp. V. Pablo; O. alba-carba; O. robusta and O. Spp*	[82]
	Vulgaxanthin IV	R3 = leucine	*O. streptacantha; O. beta vulgaris; O. alba-carba; O. robusta Wendl, O. ficus-indica*	[78,81]
	Miraxanthin II	R3 = aspartic acid	*O. bergeriana; O. ficus indica*	[12]
Betacyanins	Betanin	R1 = R2 = H	*O. robusta Wendl, O. robusta, O. streptacantha Lemaire, O. ficus-indica, O. megacantha, O. albi-carpa, O. xoconostle*	[8,12,78,82]
	*iso*-Betanin	R1 = R2 = H	*O. robusta Wendl, O. robusta, O. streptacantha Lemaire, O. ficus-indica, O. xoconostle*	[8,78,82]
	Betanidin	R = H	*O. robusta Wendl, O. robusta, O. streptacantha Lemaire, O. ficus-indica, O. megacantha, O. xoconostle*	[8,12,78,82]
	Gomphrenin i	R1 = R2 = H	*O. robusta Wendl, O. robusta, O. ficus-indica*	[78,82]
	Phyllocactin	R1 = malonyl R2 = H	*O. xoconostle*	[8,12,81,82]

### 4.2. Cactus in Health and Disease

Diverse benefits of cactus extracts and cactus compounds have been suggested by the traditional medicine uses (see below). Meanwhile, these benefits have progressively received a scientific basis thanks to numerous experimental models dedicated to the evaluation of cactus compounds to treat different diseases. Therapeutic potential has been suggested for metabolic syndrome (including diabetes type 2 and obesity), non-alcoholic fatty liver disease (NAFLD), rheumatism, cerebral ischemia, cancers, and virological and bacterial infections [83,84,85,86]. Interestingly, cactus preparations might exert preventive and therapeutic effects against alcoholism and alcohol addiction [87].

On the basis of our current knowledge of redox biology of normal and diseased cells, including cancer ones, the concept of “antioxidant” activity of phytochemicals has to be carefully evaluated. It should be recalled that action mechanisms of phytochemicals might be different according to the context. Indeed, although ROS can promote cell damage, inflammation and cancer, the so-called antioxidants, including the dietary “antioxidants” may fail to protect, and may even be dangerous for healthy cells under certain conditions. It should also be kept in mind that ROS are necessary to cells, where they play important roles, and serve the essential function to maintain the peroxide or nucleophilic tone governing all cell functions. Redox-active phytochemicals (or products from their transformation after absorption) are possibly used by cells to cause adaptive responses allowing induction of molecular defenses or block of dangerous processes, which may be different from cell to cell and from healthy to malignant cells.

### 4.3. Cactus Use in Traditional Medicine

In traditional medicine, *Opuntia ficus indica* has been used for the treatment of burns, wounds, edema, hyperlipidemia, obesity and catarrhal gastritis. Alcoholic extracts are indicated for anti-inflammatory, hypoglycemic, and antiviral purposes [84]. 

## 5. Medical Relevance of Cactus Compounds: The State of the Art

### 5.1. Experimental Models and Randomized Trials

Experiments on animal and cell models have highlighted therapeutic potentialities of cactus extracts or compounds through their impacts on key parameters involved in diseases previously targeted by traditional herbal medicines. These scientific studies and bodies of experimental proofs have strengthened the attraction of the pharmacological industry for exploring cactus as a tool to identify new natural bioactive leads and to develop new nutritional supplementations or formulations.

### 5.2. Pharmacological Potentials of Antioxidant and Antiinflammatory Effects of Cactus

*In vitro* and *in vivo* studies are convergent to conclude that *Opuntia ficus indica* extracts exhibit antioxidant and anti-inflammatory properties. The models and conditions, in which these cactus compound properties are highlighted, obviously support that they may be subject to further pharmacological exploration and development.

#### 5.2.1. *In Vitro* Studies (on Intact Cells)

Oxidative stress and inflammation are involved in numerous diseases. Many studies support the fact that many dietary redox active/antioxidant and anti-inflammatory phytochemicals are promising compounds to prevent oxidative and inflammatory mechanisms taking place in many pathological states. In human intestinal epithelial cancer cells (Caco-2) stimulated by IL-1β, co-treatment with indicaxanthin (a pigment from the edible fruit of *Opuntia ficus-indica*) prevents activations of NOX-1 and NF-kB and attenuates the rise in inducible NO synthase [88]. These data suggest that cactus dietary pigments may directly influence intestinal inflammatory mechanisms [88]. In human chondrocyte cultures stimulated with IL-1β, lyophilized extracts of *Opuntia ficus-indica* cladodes reduce the production of key molecules usually released upon chronic inflammation such as nitric oxide (NO), glycosaminoglycans, prostaglandin-E2 (PGE-2) and reactive oxygen species [89]. For this reason, lyophilized extracts of *Opuntia ficus indica* cladodes might have a pharmacological interest in preventing cartilage alterations and in treating joint disease. On human umbilical vein endothelial cells (HUVECs), non-cytotoxic micromolar concentrations of betalain (a pigment of *Opuntia ficus-indica* purified from fresh pulp of cactus pear) decrease the expression of cell adhesion molecules such as ICAM-1 [90]. Because it has also radical scavenging/antioxidant properties [90], betalain exhibits an interesting pharmacological profile for degenerative disorders affecting endothelial function such as atherosclerosis, atherothrombosis, low limb ischemia, and stroke. On the murine microglial cell line (BV-2), a butanol fraction (obtained from 50% ethanol extracts of *Opuntia ficus indica* and hydrolysis products) inhibits the production of NO in LPS-activated BV-2 cells *via* suppression of iNOS protein and mRNA expressions, inhibits the degradation of IκB-α, and displays peroxynitrite scavenging activity [91]. Moreover, in cultured mouse cortical cells, the butanol fraction of *Opuntia ficus indica* significantly reduces *N*-methyl-d-aspartate-, kainate-, and oxygen-glucose deprivation-induced delayed neurotoxicity [92]. These results support that *Opuntia ficus indica* might alleviate neuronal damages resulting or not from microglial activation. 

#### 5.2.2. *In Vivo* Studies (on the Whole Animals)

In a rat model of acute inflammation (pleurisy), the oral administration of indicaxanthin (mentioned above) reduces exudate size and leukocytes recruitment in the pleural cavity, as well as the protein and/or mRNA expressions of PGE-2, NO, IL-1β, iNOS, and cyclooxygenase-2 (COX2) in the recruited leukocytes [93]. In gerbils, protective effects of methanol extracts of *Opuntia ficus indica* given *per os* were also observed against neuronal damages caused by global ischemia in the hippocampal region [92].

### 5.3. Pharmacological Potentialities of Cactus Effects on Non-Alcoholic Fatty Liver Disease

Non-alcoholic fatty liver disease is a complex pathology involving oxidative stress, inflammation, and cell death. Noteworthy, when obese Zucker (*fa/fa*) rats are fed with a diet containing 4% *Opuntia ficus indica* for 7 weeks, the rats have around 50% lower hepatic triglycerides than the control group along with a reduction of hepatomegaly and biomarkers of hepatocyte injury (alanine and aspartate aminotransferases). A higher concentration of adiponectin and a greater abundance of genes involved in lipid peroxidation, lipids export and production of carnitine palmitoyltransferase-1 and microsomal triglyceride transfer proteins are observed in livers from cactus-treated animals. Furthermore, rats fed with cactus have a lower postprandial serum insulin concentration and a greater phosphorylated protein kinase B (pAkt):Akt ratio in the postprandial state [94]. Altogether, data obtained in obese Zucker (*fa/fa*) rats fed with *Opuntia ficus indica* support that cactus consumption attenuates hepatic steatosis, a pathology currently under the radar screen of the pharmacological industry.

### 5.4. Pharmacological Potentials of Antimicrobial Activities of Cactus

*Campylobacter* is one of the most common agent causative of food-borne bacterial gastroenteritis in the humans. Epidemiological studies reveal that consumption of poultry products represents an important risk factor of this disease. Noteworthy, the extracts of *Opuntia ficus indica* have marked bactericidal effects on the growth of *Campylobacter jejuni* and *Campylobacter coli*. Moreover, adherence of *Campylobacter* to Vero cells is strongly reduced [95]. 

Antimicrobial activities of methanolic, ethanolic, or aqueous extracts of *Opuntia ficus indica* have also been studied on *Vibrio cholerae*, indicating that the methanolic extract was the most efficient [96]. This extract causes membrane disruption, leading to increased membrane permeability and consequent marked decreases in pH and ATP. 

Altogether, these data obviously support a pharmacological interest of *Opuntia ficus indica* preventing food contamination by *Campylobacter* and *Vibrio cholerae* and in treating gut tract disorders associated with these microorganisms.

### 5.5. Pharmacological Potentials in Targeting Alcoholism with Cactus Extracts

Several studies have evaluated the benefits of *Opuntia ficus indica* against symptoms of alcohol hangover in humans. The cause of severity of the alcohol hangover can be, at least in part, inflammation and disruption of lipid metabolism homeostasis. In the rat, the effect of mucilage obtained from cladodes on the healing of ethanol-induced gastritis seems correlated with a (re)stabilization of plasma membranes in damaged gastric mucosa. Molecular interactions between mucilage monosaccharides and membrane phospholipids (mainly phosphatidylcholine and phosphatidylethanolamine) may represent the molecular basis for changes in the functions of membrane-attached proteins observed during the healing process consecutive to chronic gastric mucosal damages [97]. Moreover, in humans, an extract of the *Opuntia ficus indica* plant has been reported to reduce the symptoms of alcohol hangover like nausea, dry mouth, and anorexia [98]. 

### 5.6. Side Effects Caused by Cactus Compounds

Little information is currently available on cactus side effects. To date, a low colonic obstruction has been attributed to the consumption of *Opuntia ficus indica* seeds [99].

## 6. Conclusions

During the last decade, growing interest in cactus has resulted in a large number of scientific papers describing the composition and/or the bioactivity of a whole extract or a specific purified cactus compounds. Beside the compound contents of *Optunia ficus-indica*, this review has also devoted a special effort to account for the biological activities of the different parts of the cactus plant (summarized in Table 8). Interestingly, data from several human trials or rodent experiments show that cladodes and fruits extracts are the cactus preparations the most widely tested for their biological activities. Accordingly, as potential metabolic regulators, cactus extracts reveal beneficial effects on the metabolisms of both lipid and glucose, which bode well for the treatment of human metabolic disorders including diabetes and obesity. On the other hand, antioxidant and anti-inflammatory properties of cactus pear and cladodes need to be explored in depth to better understand biological activities and preventive potentials exhibited against several age-linked diseases by polyphenols and flavonoids abundant in cactus pear. At the nutritional level, cactus may be used as an alternative source of natural colors and nutriments, *via* supply in betalains, aminoacids, sugars, proteins and vitamins. The latter compounds offer a high nutritional value to the food industry for which the development of a real cactus-sourcing branch is awaited.

**Table 8:** Major bioactive effects of cactus preparations in different experimental models.

Biological Activity	Source of Cactus Products	*In Vivo* and *in Vitro* Models	References
Hypolipidemic and Hypocholesterolemic	Cladodes powder	Rats	[14]
	Cladodes (Glycoproteine)	Mice	[100]
	Seeds powder and seeds oil	Rats	[53]
Anti-diabetic	Capsule: cladode and fruit skin extract	Human	[101]
	Cactus powder in capsule	Human (Man and women)	[102]
	Aqueous extract of the cladode and fruit and mixture	Rats	[103]
	Cladode and fruit skin extract capsule	Man	[104]
Hypoglycemic	Polysaccharide extract from the cladode	Rats	[105]
	Extract powder racket after drying	Rats	[106]
Anti-Inflammatory	Indicaxanthin, from fruit	Human intestinal epithelial cell line (Caco-2 cells) stimulated by cytokine IL-1b	[88]
	Lyophilized extracts of cladodes	Human chondrocyte cultures stimulated with IL-1β	[89]
	Indicaxanthin from Cactus Pear Fruit	Rat Pleurisy obtained by injection of 0.2 ml of λ-carrageenin into the pleural cavity	[93]
	Methanol extract of cactus stems (active substance: β-sitosterol)	Mice (male)	[107]
	Methanolic extracts of prickly pear fruits (Betalain Indicaxanthin)	In vitro study of the interaction between purified Betalains and HOCL and human myeloperoxidase	[93,108]
Anti-Inflammatory and Antioxidant	Butanol and methanol fruit extract	In vivo studies in gerbils and In vitro studies in cultured mouse cortical cells	[92]
Antioxidant	Betalain a pigment purified from fresh pulp of cactus pear	Endothelial cells human umbilical vein (HUVEC)	[90]
	Betanin prickly pear fruit Extracts	Chemical and biological (human RBC, LDL) systems	[1]
	Ethanol extract of the stem	Chemical and biological systems (mouse splenocytes)	[22]
	Flavonoid fraction of juice of whole fruits	Rats	[18]
	Glycoprotein (90 kDa) isolated from Opuntia ficus-indica var. saboten MAKINO	Mice induced by Triton WR-1339	[100]
	Cactus pear fruit	Healthy humans (10 women and 8 men) supplemented with cactus pear or Vit C	[109]
	Quercetine ether 3-O-méthyl isolated from Opuntia ficus-indica var. saboten	Primary cultured rat cortical cells	[17]
Antimicrobial	Methanol extract of cladode	Bacteria: *Campylobacter jejuni* and *Campylobacter coli*	[95]
	Methanolic, ethanolic, and aqueous extracts of cladode	Bacteria: *Vibrio cholerae*	[96]
	Hexane extracts from flowers	Bacteria: *Staphylococcus aureus*, *Escherichia coli*, *Pseudomonas* *aeruginosa* and *Bacillus subtilis*	[110]
	Aqueous and alcoholic extracts of cladode	Bacteria: *Proteus mirabilis*	[111]
